# The Expression of Laminin-5 in Malignant Lesions of the Oral Cavity and Its Association With the Aggressiveness of the Tumor

**DOI:** 10.7759/cureus.85406

**Published:** 2025-06-05

**Authors:** Deepak K Gupta, Nitin K Sharma, Neena Chaudhary, Usha Agrawal, Vishwadeep Singh

**Affiliations:** 1 Otolaryngology - Head and Neck Surgery, Vardhman Mahavir Medical College and Safdarjung Hospital, New Delhi, IND; 2 Pathology, ICMR-National Institute of Pathology, Safdarjung Hospital Campus, New Delhi, IND

**Keywords:** carcinoma buccal mucosa, carcinoma tongue, laminin 5, malignant lesions of oral cavity, oral squamous cell carcinomas

## Abstract

Oral squamous cell carcinoma (OSCC) constitutes a major subset of head and neck cancers, with significant variability in clinical outcomes. Laminin-5, a key extracellular matrix protein involved in cell adhesion and invasion, may serve as a biomarker for tumor aggressiveness. This cross-sectional study involved 30 histologically confirmed OSCC patients who underwent surgical excision at Safdarjung Hospital, New Delhi. Immunohistochemical staining was used to assess laminin-5 expression, which was then correlated with tumor thickness, differentiation, TNM staging, and metastasis. Laminin-5 positivity was observed in 83.4% of cases. A statistically significant association was found between laminin-5 expression and tumor differentiation (p = 0.027), with the highest mean expression seen in moderately differentiated tumors (65.24%). No significant correlation was noted between laminin-5 expression and tumor stage, nodal status, metastasis, or tumor thickness. These findings suggest that laminin-5 may serve as a useful histological marker of tumor differentiation in OSCC and could aid in evaluating tumor behavior and prognosis.

## Introduction

Head and neck squamous cell carcinoma (HNSCC) ranks as the sixth most prevalent cancer globally, with over 550,000 new cases and approximately 300,000 deaths reported each year. Squamous cell carcinoma (SCC) constitutes 90% of all head and neck malignancies, with oral carcinomas accounting for about 85% of these cases. The oral cavity includes several anatomical sub-sites such as the tongue, floor of the mouth, gingiva, palate, and buccal mucosa. The pattern of site involvement varies across regions; in Western countries, the tongue is most frequently affected, whereas in many Asian populations, there is a buccal mucosa predominance, which is attributed to the widespread use of betel quid and tobacco [1].

In India, oral cancer is the leading malignancy among men and the third most common among women, with a male-to-female ratio of 1.7:1. It contributes to nearly 40% of all cancer cases and remains a significant health burden, particularly in resource-limited settings. Oral squamous cell carcinoma (OSCC) frequently presents with early lymphatic spread and is most common in individuals in their fifth and sixth decades, often linked to tobacco and alcohol use. Delayed diagnosis - often due to misdiagnosis or lack of awareness - contributes to its poor prognosis. The five-year survival rate for OSCC ranges from 40 to 50% [2], and despite improvements in medical interventions, both incidence and mortality rates remain high.

Traditional prognostic methods rely on TNM staging, tumor grade, and depth of invasion. However, these clinical and histopathological parameters often fail to fully reflect the tumor’s biological behavior. Molecular biomarkers are increasingly recognized for their potential role in diagnosis, treatment stratification, and prognosis. Among them, epidermal growth factor receptor (EGFR), connexins, and laminins have shown promise. EGFR plays a critical role in tumor cell growth, survival, and metastasis. Connexin-43, a component of gap junctions, has been identified as a prognostic marker in several cancers. Laminin-5, a key extracellular matrix protein of the basal lamina, supports epithelial cell adhesion and tissue integrity. Its interaction with type VII collagen and degradation by tumor-associated proteases is implicated in cancer cell invasion and metastasis [3,4].

Given that tumor thickness, nodal status, and histological differentiation are established predictors of aggressiveness in OSCC, the evaluation of laminin-5 expression in relation to these parameters may enhance prognostic accuracy. This study aims to investigate the expression of laminin-5 in OSCC and its association with tumor thickness, differentiation, T and N staging, and pathological lymph node involvement, thereby exploring its utility as a potential tumor marker for oral cancers.

## Materials and methods

Study design and setting

This cross-sectional study was conducted at the Department of Otorhinolaryngology, Safdarjung Hospital, in collaboration with the ICMR-National Institute of Pathology (Safdarjung Campus), New Delhi. The study population comprised patients of any age and sex presenting to the outpatient department with biopsy-proven OSCC, who were planned for surgery as the primary treatment modality. Patients were recruited over a period of 18 months.

Sampling

The sample size was determined based on previous research by Shweta et al., which reported positive laminin-5 expression in 16% of cases. Since data on its association with T and N staging, lymph node metastasis, and tumor thickness were lacking, this prevalence estimate was used for calculation [5]. Using a power of 80%, a significance level of 5%, and a finite population size of 40, the minimum required sample size was estimated to be 22. To account for possible exclusions due to inadequate staining, missing data, or dropouts, and to enhance the statistical power for subgroup comparisons, we rounded up and included 30 patients in the study.

Inclusion and exclusion criteria

Inclusion criteria included all OSCC patients undergoing surgical excision and reconstruction, with or without neck dissection. Exclusion criteria were as follows: evidence of distant metastasis, prior head and neck irradiation or surgery, pregnancy, and bleeding diathesis.

Data collection

After obtaining informed consent, detailed clinical histories were taken, with emphasis on known risk factors such as tobacco use and smoking. A thorough clinical examination was conducted to assess the tumor site, size, and extent, as well as cervical lymph node involvement for staging purposes. Baseline investigations included hematological tests, chest radiograph, and abdominal ultrasound to evaluate for distant metastasis. For local and regional staging, neck ultrasonography and contrast-enhanced CT (CECT) from the skull base to the thoracic inlet were performed.

Diagnosis and tumor grade were confirmed by biopsy of the primary lesion. All patients underwent surgical excision of the tumor, with or without neck dissection and reconstruction. Neck dissection specimens were categorized into anatomical levels I to V and sent for histopathological evaluation. The pathologist, blinded to the clinical and imaging data, evaluated the excised tissue for laminin-5 expression, tumor thickness, and histological differentiation. Immunohistochemical staining was used to detect laminin-5, with positive expression identified as a brown reaction product. Both the tumor tissue and the adjacent tumor-negative mucosa were examined for qualitative and intensity-based expression patterns. The laminin-5 expression results were subsequently correlated with clinical and pathological parameters, including tumor size (T stage), nodal involvement (N stage), lymph node metastasis, tumor differentiation, and tumor thickness.

Statistical analysis

All data were entered in Microsoft Excel (Microsoft Corporation, Redmond, WA) and analyzed using SPSS Statistics version 21.0 (IBM Corp., Armonk, NY). Continuous variables were expressed as mean, median, standard deviation (SD), and interquartile range (IQR). The Shapiro-Wilk test revealed that the data were not normally distributed. Fisher's exact test was used for comparing categorical variables across groups. Continuous variables were compared using the Mann-Whitney test for two groups and the Kruskal-Wallis test for comparisons across more than two groups. A p-value <0.05 was considered statistically significant.

## Results

A total of 30 patients with histopathologically confirmed OSCC were enrolled over 18 months. Patients underwent comprehensive clinical assessment and surgical excision of the primary lesion, with or without neck dissection. Tumor samples were analyzed for laminin-5 expression, and the findings were correlated with clinical and pathological parameters.

The mean age of the study population was 47.1 ± 11.15 years (range: 26-70 years). There was a male predominance, with 80% (n = 24) of participants being male (M:F ratio = 4:1). The most common tumor site was the lateral border of the tongue (50%), followed by the buccal mucosa (36.7%). The majority of cases presented with T2 (43.3%) and T3 (40%) tumors, and 60% (n = 18) were classified as N0 in nodal staging. Pathological metastasis was identified in 40% of cases. Clinically, 36.7% were in Stage III, followed by Stage II (26.7%) and Stage IVB (20%). Laminin-5 expression was positive in 25 cases (83.4%) and negative in five cases (16.6%). The mean expression was 50.16 ± 32.76%, with a median of 60% (range: 0-100%). Laminin-5 expression intensity was categorized as moderate in 46.7%, mild in 26.7%, severe in 10%, and nil in 16.6%.

The association between laminin-5 expression and various clinical and pathological parameters is summarized in Table 1. Laminin-5 was found to be positive in 83.4% (25/30) of OSCC cases. No statistically significant association was observed between laminin-5 status and gender (p = 1.000), T-stage (p = 0.937), N-stage (p = 0.301), clinical staging (p = 0.180), or pathological metastasis (p = 1.000). However, a significant correlation was identified with tumor differentiation (p = 0.027), with positive laminin-5 expression most frequently observed in moderately differentiated tumors, followed by well-differentiated tumors, and absent in dysplastic or hyperplastic mucosa.

**Table 1:** Association of laminin-5 status with gender, TNM staging, clinical stage, tumor differentiation, and metastasis in OSCC patients (N = 30) ^*^P<0.05; ^#^Fisher’s exact test applied OSCC: oral squamous cell carcinoma

	Laminin-5 status		Total	P-value
	Positive	Negative		
Gender				
Male	20	4	25	1.00^#^
Female	5	1	6	
TNM staging (T)				
T1	3	1	4	0.94^#^
T2	11	2	13	
T3	10	2	12	
T4A	1	0	1	
TNM staging (N)				
N0	15	3	18	0.30^#^
N1	4	2	6	
N3B	6	0	6	
TNM staging (M)				
Present	10	2	12	1.00^#^
Absent	15	3	18	
Clinical staging				
Stage I	3	1	4	0.18^#^
Stage II	8	0	8	
Stage III	7	4	11	
Stage IVA	1	0	1	
Stage IVB	6	0	6	
Tumor differentiation				
Hyperplastic mucosa	0	1	1	0.03^*#^
Well differentiated	2	0	2	
Moderately differentiated	21	3	24	
Poorly differentiated	2	0	2	
High-grade dysplasia	0	1	1	

**Figure 1:**
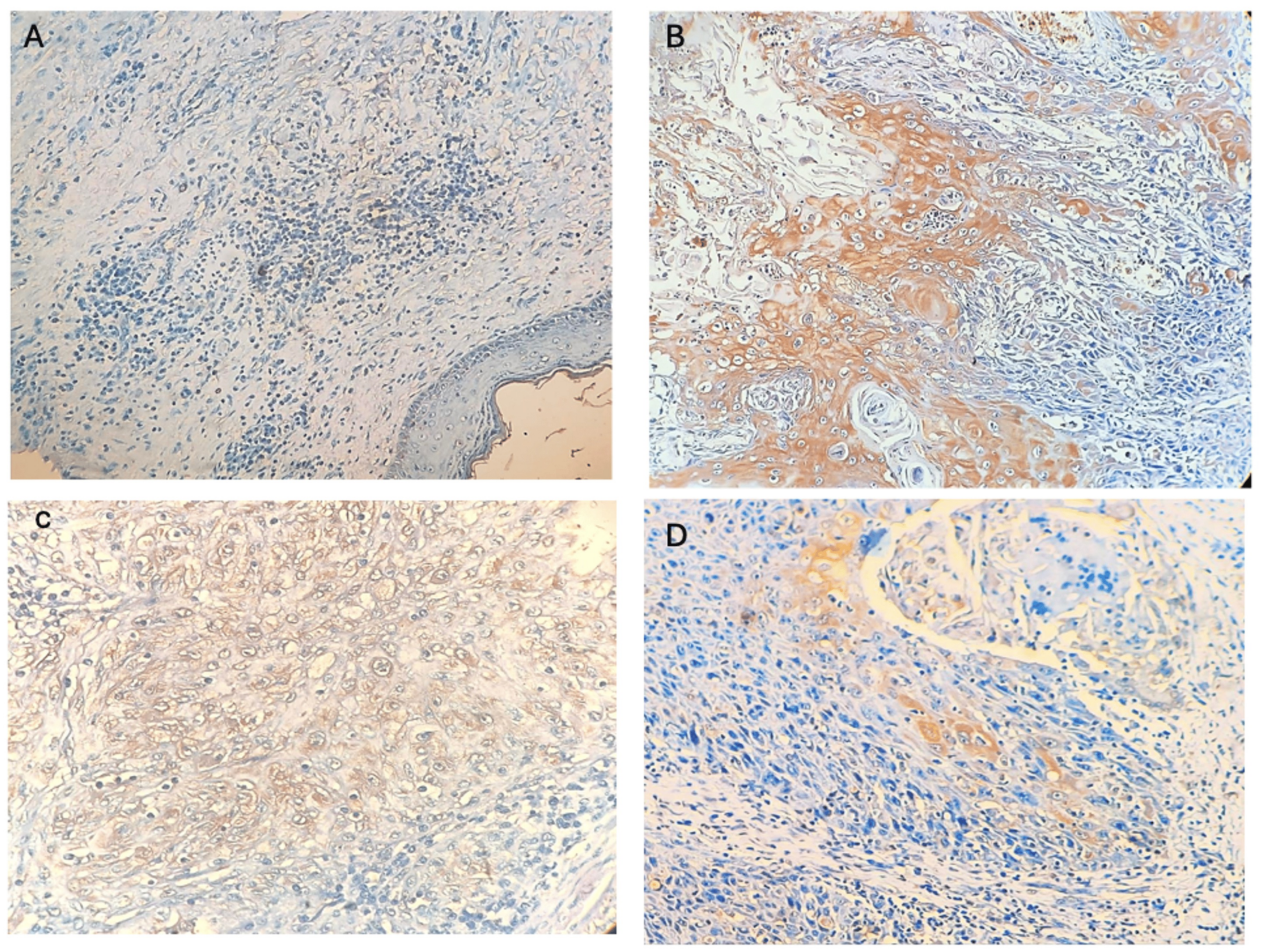
Tumor differentiation A: Normal cells showing negative expression for laminin-5. B: Well-differentiated tumor shows cytoplasmic expression for laminin-5. C: Moderately differentiated SCC shows laminin-5 expression in all the tumor cells. D: Poorly differentiated SCC showing moderate expression (5% tumor cells) of laminin-5 SCC: squamous cell carcinoma

The corresponding expression levels and their distribution across clinical variables are further detailed in Table 2. While the mean laminin-5 expression was slightly higher in females (53.33%) than in males (49.37%), this difference was not statistically significant (p = 0.853). Across clinical stages, the highest mean expression was observed in Stage I (70.0%), with decreasing trends noted in Stage IVB (53.33%), though this did not reach statistical significance (p = 0.941). Tumor differentiation again showed a significant association with laminin-5 expression (p = 0.030), with the highest mean expression in moderately differentiated tumors (65.24%), followed by well-differentiated (50.00%) and poorly differentiated tumors (17.50%) (Figure 1). Expression was also higher in metastatic cases (62.00%) compared to non-metastatic cases (59.00%), but the difference was not statistically significant (p = 0.783).

**Table 2:** Comparison of mean laminin-5 expression across gender, clinical staging, tumor differentiation, and pathological metastasis in OSCC patients (N = 30) ^*^P<0.0016 (Bonferroni correction); ^#^Kruskal-Wallis Test applied; ^$^Mann-Whitney U applied OSCC: oral squamous cell carcinoma; SD: standard deviation

		Laminin-5 expressed		P-value
		Mean (SD)	Median (SD)	
	Gender			
Male		49.37 (±30.62)	60 (±80)	0.85^$^
Female		53.33 (±37.71)	60 (±50)	
	Clinical staging			
Stage I		70.0 (±10.0)	65 (±45)	0.94^#^
Stage II		60.63 (±29.81)	70 (±20)	
Stage III		61.43 (±29.68)	30 (±80)	
Stage IVA		60.00 (±0)	60 (±0)	
Stage IVB		53.33 (±27.32)	50 (±50)	
	Tumor differentiation			
Well differentiated		50.00 (±28.28)	50 (±40)	0.0007^*#^
Moderately differentiated		65.24 (±22.94)	70 (±40)	
Poorly differentiated		17.50 (±17.68)	17.5 (±25)	
	Pathological metastasis			
Present		62.00 (±23.48)	60 (±55)	0.78^$^
Absent		59.00 (±28.04)	60 (±60)	

The intensity of laminin-5 staining, as illustrated in Table 3, revealed a predominantly moderate pattern (46.7%) across the study group, followed by mild (26.7%) and severe (10%) staining; 16.6% of cases showed no laminin-5 expression. Severe intensity was observed exclusively in advanced cases (Stage IVB) and those with metastasis, suggesting a trend toward higher expression in aggressive tumors, though these associations did not achieve statistical significance (p = 0.055 and p = 0.069, respectively). No significant association was found between laminin-5 intensity and tumor differentiation (p = 0.896), although moderate expression was most common in moderately differentiated tumors.

**Table 3:** Association of laminin-5 expression intensity with clinical stage, tumor differentiation, and pathological metastasis in OSCC patients (N = 25) ^#^Fisher’s exact test applied OSCC: oral squamous cell carcinoma

	Laminin-5 intensity			Total	P-value
	Mild	Moderate	Severe		
Clinical staging					
Stage I	2	1	0	3	0.055^#^
Stage II	1	7	0	8	
Stage III	3	4	0	7	
Stage IVA	1	0	0	1	
Stage IVB	1	2	3	6	
Tumor Differentiation					
Well differentiated	1	1	0	2	0.90^#^
Moderately differentiated	6	12	3	21	
Poorly differentiated	1	1	0	2	
Pathological metastasis					
Present	2	5	3	10	0.07^#^
Absent	6	9	0	15	

Laminin-5 expression was analyzed in relation to tumor thickness. The mean tumor thickness was higher in cases that expressed laminin-5 (1.08 ± 0.49 cm) compared to those that did not (0.92 ± 0.41 cm), although this difference was not statistically significant (p = 0.494). Similarly, when examined across different intensities of laminin-5 staining, the mean tumor thickness increased with staining severity-ranging from 0.90 cm in mild expression to 1.27 cm in severe expression, but this trend also did not reach statistical significance (p = 0.431).

## Discussion

OSCC represents a heterogeneous group of malignancies with distinct clinical behaviors, treatment responses, and prognostic outcomes based on anatomical site, differentiation, and tumor biology. These differences also necessitate varied approaches in functional and cosmetic preservation. In recent years, advances in molecular oncology have significantly enhanced our understanding of the genetic and molecular alterations underlying tumor progression and invasion. Among these, laminin family proteins have emerged as prominent molecular markers, particularly laminin-5 [6-8]. It is a high molecular weight glycoprotein of the extracellular matrix; it constitutes a key component of the basal lamina and plays a vital role in cellular adhesion, migration, and differentiation. It has been frequently detected at the invasive front of carcinomas, implicating its involvement in tumor progression, especially in SCC. Mechanistically, laminin-5 interacts with integrin α6β4 and EGFR, triggering the activation of phosphatidylinositol-3-kinase (PI3K), a signalling pathway that governs tumor proliferation, invasion, and apoptosis evasion. Its activation is associated with more aggressive tumor behavior and poor prognosis, making laminin-5 a potential target for anti-tumor therapies. However, a complete understanding of its role in tumor biology is essential before it can be therapeutically exploited [9-13].

This study assessed laminin-5 expression in 30 OSCC patients, all of whom underwent clinical, radiological, and pathological evaluation. The mean age was 47.1 years (range: 26-70), with a male-to-female ratio of 4:1. No significant association was found between laminin-5 expression and age or gender. The most commonly affected site in our cohort was the lateral border of the tongue (50.0%), followed by the buccal mucosa (36.7%). This pattern contrasts with findings from Tandon et al., who reported a higher incidence of buccal mucosa involvement in their cohort (31.47%) [14]. This regional variation may be attributed to differing risk factor profiles, such as tobacco chewing practices prevalent in certain populations. When laminin-5 expression was correlated with tumor thickness, T-stage, N-stage, and clinical stage, no significant associations were identified. Our findings are in line with those of Ono et al., who similarly did not find a statistically significant relationship between laminin-5 expression and TNM staging or lymph node involvement [15].

A notable finding in the present study was the statistically significant association between laminin-5 expression and tumor differentiation. The expression was observed in 100% of both well- and poorly differentiated OSCC cases, and in 88% of moderately differentiated tumors. None of the hyperplastic or high-grade dysplasia cases showed laminin-5 expression. This relationship between laminin-5 positivity and tumor differentiation was found to be statistically significant (p = 0.026). Furthermore, the mean laminin-5 expression was highest in moderately differentiated tumors and lowest in poorly differentiated ones, and this difference was also statistically significant (p = 0.03). These findings suggest a potential role for laminin-5 as a histopathological marker of tumor differentiation in OSCC.

Our results are consistent with previous studies. Abdel Naser et al. demonstrated laminin-5 immunopositivity in all OSCC cases, with higher expression in well-differentiated tumors than in moderate and poorly differentiated ones, indicating a strong correlation between laminin-5 expression and tumor differentiation [16]. Similarly, Ono et al. found that laminin-5 expression intensity correlated with infiltrative growth patterns and poorer differentiation. They concluded that higher expression levels were indicative of increased invasiveness and a worse prognosis [15]. In contrast, Yellapurkar et al. observed intense cytoplasmic laminin-5 staining in 75% of poorly differentiated lesions, while mild to moderate expression was seen in well- and moderately differentiated cases. Their study emphasized that poorly differentiated lesions exhibited increased cytoplasmic laminin-5 expression, signifying enhanced invasiveness. They also reported linear staining at the tumor-connective tissue interface in well-differentiated lesions, but no such staining in moderately and poorly differentiated cases. Their findings suggest that increased cytoplasmic expression of laminin-5, along with the absence of linear interface staining, may indicate invasive potential and margin involvement [5].

In our study, no statistically significant correlation was observed between laminin-5 status, expression intensity, and clinicopathological parameters such as sex, tumor stage, metastasis, or tumor thickness. Despite the lack of statistically significant associations with these routine parameters, the consistent relationship with tumor differentiation highlights the potential utility of laminin-5 as a histological biomarker of tumor behavior.

Overall, the findings from this study, in conjunction with prior literature, endorse the role of laminin-5 as a useful adjunct in assessing tumor differentiation and possibly the aggressiveness of OSCC. This study offers valuable region-specific data on laminin-5 expression in OSCC patients, with objective immunohistochemical evaluation by a blinded pathologist and a combination of qualitative and quantitative analysis, enhancing the reliability of findings. However, it is limited by a small sample size, the absence of inter-observer validation, and a lack of multivariate analysis to control for confounding variables. Future research should involve larger, multicenter cohorts, incorporate additional biomarkers such as Ki-67 or EGFR, and examine the biological relevance of laminin-5 localization patterns to develop more comprehensive prognostic models.

## Conclusions

Laminin-5 was expressed in a majority of OSCC cases and demonstrated a statistically significant association with tumor differentiation. While it did not correlate with tumor stage, nodal status, metastasis, or thickness, its consistent link with histological grading highlights its potential utility as a supportive biomarker for assessing tumor differentiation. Further large-scale studies are warranted to clarify its prognostic value and explore its role in targeted therapeutic strategies.
